# Co-application of gibberellin and copper sulphate reduces number of seeds as innovative technique for alleviating alternate bearing in Balady mandarin

**DOI:** 10.1038/s41598-025-22663-2

**Published:** 2025-10-27

**Authors:** Ayman E. A. Shaban, Mohammed I. M. El-Banna, Ahmed A. Rashedy

**Affiliations:** Pomology department, Faculty of Agriculture, Cairo University, Cairo, Egypt

**Keywords:** Citrus reticulate, Fruit set, Yield, Fruit quality, Biennial bearing, Plant development, Plant hormones, Plant physiology, Plant sciences

## Abstract

Seeds are regarded as a source of gibberellins (GA) which promote fruit set, fruit growth and plant growth. However, trees with more-seeded fruits tend to alternate bearing (AB) due to high gibberellins supplies. So, this investigation aimed to reduce number of seeds via spraying with 1000 ppm GA or copper sulphate (0.1% CuSO_4_) or 1000 ppm GA + 0.1% CuSO_4_ twice during flowering as a new technique to alleviate AB in Balady mandarin tree. Generally, individual spray of GA or CuSO_4_ reduced number of seeds per fruit and AB. Also, the combined application of GA + CuSO_4_ recorded the lowest significant number of seeds by 35.48% & 37.22% and fruit set by 27.6% & 25%, meanwhile, it recorded the highest increase percentage of fruit weight (32.2% & 37.2%) and marketable yield (17.7% & 28.17%) compared to the control in the two On year seasons. In addition, this treatment recorded the lowest concentration of endogenous GA in leaves of mandarin trees (7.6 & 7 µg/g F.W.) with the highest reduction percentage of 16.48% & 22.2% in comparison to the control (9.1 & 9 µg/g F.W.) in both seasons, respectively. Thus, this treatment reduced endogenous GA levels by 16.48% & 22.2% and AB index by 55.4% & 50.7% in both seasons. Furthermore, in the following two Off seasons, GA + CuSO_4_ improved fruit weight by 2.4% & 16.7%, number of fruits by 76.7% & 97.7%, and yield by 58.3% & 107.7%, in both seasons, respectively. Accordingly, GA + CuSO_4_ proved to be efficient treatment which effectively reduced endogenous GA in leaves of mandarin trees, produced low-seeded fruits (high quality fruits), reduced AB index and improved On year yield via increasing fruit weight and the following Off year yield via increasing fruits number per tree.

## Introduction

Mandarin (*Citrus reticulata*, Blanco) ranks as one of the most popular fruits among citrus varieties in Egypt, due to its high yield, fresh consumption, easy peeling and acceptable juice quality as well as nutritional value^1^. The planted area of mandarins amounted to about 24.6% of total area of citrus area in Egypt, with a production of 1,174,895 tons in 2022^2^. The mandarin fruits are beautiful in shape and easy peeling and considered a good source of vitamins (C, B, A), antioxidant compounds and minerals^3^.

Alternate bearing (AB) refers to a phenomenon which means that trees produce a large yield of small-sized fruit in on year followed by a low yield in the following Off year. AB has been observed in different deciduous trees^4^ and evergreen trees^5–7^. Although AB is non-common in seedless citrus fruit such as navel orange, grapefruit, and lemon fruit but it is common in more-seeded fruits such as mandarin^5,6^ and orange^8^ with exception for more-seeded fruits Satsuma mandarin which had a male and female sterility^9^. Also, seeds inside the developing fruits exert strong inhibitory effect on flower bud production^10^. In alternating citrus cultivars, final yields rely more on the number of flowers and initial fruit set, complicating natural fruit thinning^9^. In alternate-bearing fruit cultivars, high fruit load in On year is thought to negatively inhibit flower induction and development in the following Off year number^6^. Balady mandarin is known for its alternate bearing tendency^7^ and contains approximately 20 seeds per fruit^11,12^. Fruit growth during On year, produces different substances such as gibberellin acid (GA) which is known as inhibitor of flowering in citrus^13^. Seeds contain relatively large amounts of hormones like IAA (Indole acetic acid) and GA that may act together or separately to inhibit floral induction in perennial fruit trees^14^. In recent study, higher mandarin yield in On year was accompanied with higher endogenous GA levels during flower bud induction (winter) which leads to reducing flowering in the following (Off year) season^5^.

Fruit thinning is one of the most common used horticultural practices for improving fruit quality specially when there is a high fruit load^12,15,16^ and for maintaining tree performance and partitioning of photosynthetic resources^17^. Early thinning provides more nutrients, photosynthetic resources and carbohydrates for the remaining fruits. Chemical thinning is more cost-effective and saves time compared to hand thinning. GA and CuSO_4_ are commonly used for flower thinning through inducing parthenocarpy and inhibiting ovules fertilization^18–21^. Various efforts have been undertaken to minimize citrus fruit seeds. Application of 10 ppm GA used at anthesis negatively affects fertilization either by improving the abortion of ovules or reducing the growth of pollen tubes in Clementine mandarin flowers under cross-pollination conditions^22^. More recently, application of 50 ppm GA during the flower induction period (three times during December) of Orri Mandarin greatly reduce flowers number^6^.

Copper sulphate treatment considers another chemical thinning materials which also have been used to reduce number of seeds in citrus fruits as fruit quality index. CuSO_4_ is the preferred source for Cu because it is of low cost compared to chelated forms and is the primary used form in physiological studies^23^. Also, application of 25 mg L^−1^ CuSO_4_ at full bloom to mandarin trees significantly reduced the average number of seeds per fruit^23^. Utilizing sulphur-based products reduced the in vitro pollen germination of the Fortune mandarin^18^. Also, application of CuSO_4_ at full bloom to the entire trees significantly reduced the average number of seeds per fruit^24^. CuSO_4_ inhibited pollen tube growth of Nadorcott mandarin flowers by 94–100%^25^. Combined application of GA and CuSO_4_ was used to reduce number of seeds and increase percentage of seedless fruit in Afourer mandarin^18,21^. Treatment of CuSO_4_ + GA increased floral style elongation in mandarin which resulted in longer time for pollen tubes to reach the ovules, which resulted in reducing fertilization^21,22^. Therefore, the aim of the present study was to investigate the impact of individual and combined application of GA and CuSO_4_ for reducing number of seeds per fruit during the On year season of Balady mandarin trees and monitoring their cumulative impact in the following Off year as a novel tool for mitigating AB in Balady mandarin trees.

## Materials and methods

### Experimental site

The experiments were carried during two On year seasons: the first On year season was in 2021 and the second On year season was in 2022. The cumulative effect was monitored in the first Off year season in 2022 and in the second Off year season in 2023. The treatments were carried only in the On year season. For the cumulative effect in the following two Off year seasons; fruiting, fruit yield and AB were recorded. The present study was carried out at a private orchard at El-Nagah district (30°41’42"N and 30°23’16"E, elevation 9 m), El-Beheira Governorate, Egypt.

### Plant material

Twelve years-old Balady mandarin (*Citrus reticulate* Blanco) trees were budded on sour orange (*Citrus aurantium* L.) rootstock and spaced 4 × 4 m apart were selected for this study. Trees were grown in sandy soil under drip irrigation system. All trees received the horticultural practices (irrigation, fertilizers, pruning, weed control, pest control …etc) according to recommendation of Agriculture Ministry. Mandarin trees grown in open field condition (cross-pollination) with no other citrus tree species planted among them.

### Treatments

The experiment included four treatments, control (untreated trees), 1000 ppm GA_3_, 0.1% CuSO_4_ and 1000ppm GA_3_ + 0.1% CuSO_4_. All materials were used for foliar spraying twice during the flowering stage at 50% of full bloom and at full bloom. The spraying was carried in the morning using 5 L solution per tree with 0.1% tween 80 as a surfactant. The treatments were applied on 48 trees, thus the experiment included three replicates/treatment and four trees were used per replicate.

### Measurements

Twelve branches (3 branches in each direction) from each tree were labelled in early March to record the following parameters:

#### Fruit set%

Fruit set means that only the flowers that developed into fruitlets. Four main branches in each replicate were marked and final fruit set was determined at August by dividing fruit set by the total number of flowers per brunch^26^ as follows.

$$\:\text{F}\text{r}\text{u}\text{i}\text{t}\:\text{s}\text{e}\text{t}{\%}\:=\frac{\text{N}\text{u}\text{m}\text{b}\text{e}\text{r}\:\text{o}\text{f}\:\text{f}\text{r}\text{u}\text{i}\text{t}}{\text{T}\text{o}\text{t}\text{a}\text{l}\:\text{n}\text{u}\text{m}\text{b}\text{e}\text{r}\:\text{o}\text{f}\:\text{f}\text{l}\text{o}\text{w}\text{e}\text{r}\text{s}}\:\times\:100$$

#### Fruit drop %

Fruit drop means that flowers that dropped from petal fall till harvest. Four main branches in each replicate were marked, and the number of fruits was recorded twice, 7 days after fruit set and at harvest date. Consequently, the fruit drop % was recorded by dividing total dropped fruit by the total flowers number^26^ as the following.

$$\:\text{F}\text{r}\text{u}\text{i}\text{t}\:\text{d}\text{r}\text{o}\text{p}\:{\%}\:=\:\frac{\text{N}\text{u}\text{m}\text{b}\text{e}\text{r}\:\text{o}\text{f}\:\text{r}\text{e}\text{t}\text{a}\text{i}\text{n}\text{e}\text{d}\:\text{f}\text{r}\text{u}\text{i}\text{t}\:\text{a}\text{t}\:\text{h}\text{a}\text{r}\text{v}\text{e}\text{s}\text{t}}{\text{N}\text{u}\text{m}\text{b}\text{e}\text{r}\:\text{o}\text{f}\:\text{f}\text{r}\text{u}\text{i}\text{t}\:\text{s}\text{e}\text{t}\:\text{a}\text{t}\:7\:\text{d}\text{a}\text{y}\text{s}\:\text{a}\text{f}\text{t}\text{e}\text{r}\:\text{p}\text{e}\text{t}\text{a}\text{l}\:\text{f}\text{a}\text{l}\text{l}}\:\times\:100$$

### Yield component

Harvest took place when TSS/acid ratio was between 10 and 12; then total yield was determined by counting fruits number per tree and determining the average fruit weight. Fruit yield was calculated by multiplying the average fruit weight by the number of fruits^27^.

### Normal and aborted seeds %

After harvest, fruits were cut carefully, then both percentage and the average number of normal seeds per fruit were calculated. Also, percentage and the average number of aborted seeds per fruit were calculated. An aborted seed means that the seed failed to become fully developed with the embryo and cotyledons and fully developed seed coat but it contains only parts of the seed coat.

$$\text{Aborted seeds}{\%}=\:\frac{\text{N}\text{u}\text{m}\text{b}\text{e}\text{r}\:\text{o}\text{f}\:\text{a}\text{b}\text{o}\text{r}\text{t}\text{e}\text{d}\:\text{s}\text{e}\text{e}\text{d}}{\text{T}\text{o}\text{t}\text{a}\text{l}\:\text{n}\text{u}\text{m}\text{b}\text{e}\text{r}\:\text{o}\text{f}\:\text{s}\text{e}\text{e}\text{d}\text{s}}\:\times\:100$$

### Marketable and misshapen fruit yield

At harvest time, fruits that were free from irregular growth or shape with a good shape and quality were classified as marketable fruits, then their percentage was calculated. Conversely, number of misshapen fruits which appeared abnormal growth with irregular shape was calculated and their percentage was calculated as the following:

$$\text{Marketable fruit}{\%}=\:\frac{\text{N}\text{u}\text{m}\text{b}\text{e}\text{r}\:\text{o}\text{f}\:\text{m}\text{a}\text{r}\text{k}\text{e}\text{t}\text{a}\text{b}\text{l}\text{e}\:\text{f}\text{r}\text{u}\text{i}\text{t}}{\text{T}\text{o}\text{t}\text{a}\text{l}\:\text{n}\text{u}\text{m}\text{b}\text{e}\text{r}\:\text{o}\text{f}\:\text{f}\text{r}\text{u}\text{i}\text{t}}\:\times\:100$$

$$\text{Misshapen fruit}{\%}=\:\frac{\text{N}\text{u}\text{m}\text{b}\text{e}\text{r}\:\text{o}\text{f}\:\text{m}\text{i}\text{s}\text{s}\text{h}\text{a}\text{p}\text{e}\text{n}\:\text{f}\text{r}\text{u}\text{i}\text{t}}{\text{T}\text{o}\text{t}\text{a}\text{l}\:\text{n}\text{u}\text{m}\text{b}\text{e}\text{r}\:\text{o}\text{f}\:\text{f}\text{r}\text{u}\text{i}\text{t}}\:\times\:100$$

### Fruit titratable acidity %

Titratable acidity (TA) was determined by titrating 10 ml fruit juice to the end point (a stable faint pink color) with 0.1 N NaOH in presence of 1% phenolphthalein (1 g phenolphthalein in 100 ml ethanol 90%) and was expressed as a percentage of citric acid. TA determination performed from A*B equation

$$\text{A}=\:\frac{(\text{v}\text{o}\text{l}\text{u}\text{m}\text{e}\:\text{o}\text{f}\:\text{t}\text{i}\text{t}\text{r}\text{a}\text{t}\text{e}\:\times\text{n}\text{o}\text{r}\text{m}\text{a}\text{l}\text{i}\text{t}\text{y}\:\text{o}\text{f}\:\text{t}\text{i}\text{t}\text{r}\text{a}\text{t}\text{e}\:(0.1\:\text{N})}{(\text{v}\text{o}\text{l}\text{u}\text{m}\text{e}\:\text{o}\text{f}\:\text{s}\text{a}\text{m}\text{p}\text{l}\text{e}\times1000)}$$

$$\text{B}=\:\frac{\left(\text{E}\text{q}\text{u}\text{i}\text{v}\text{a}\text{l}\text{e}\text{n}\text{t}\:\text{w}\text{e}\text{i}\text{g}\text{h}\text{t}\:\text{o}\text{f}\:\text{c}\text{i}\text{t}\text{r}\text{i}\text{c}\:\text{a}\text{c}\text{i}\text{d}\:\right(64.04)\:}{\:\left(\text{v}\text{o}\text{l}\text{u}\text{m}\text{e}\:\text{o}\text{f}\:\text{s}\text{a}\text{m}\text{p}\text{l}\text{e}\:\right)}\times100$$

### Fruit total soluble solids content

Fruits were cut in half and carefully hand-squeezed in a commercial juicer. The content of total soluble solids (TSS) was determined through a handheld refractometer (model MASTER-53 S; Atago Co. Ltd., Tokyo, Japan) and expressed as °Brix.

### Fruit total sugars content

Phenol–sulfuric acid method was used to determine total sugar content Dubois^28^. Fruit pulp samples (0.25 g) were homogenized in 20 ml of 70% ethanol and then filtered. The filtrates (1 ml) were mixed with 1 ml of phenol 5% (5 g phenol in 100 ml distilled water) followed by the addition of 5 ml of sulfuric acid (98%). After 1 h, the absorbance of the colored solutions was measured at 490 nm using UV/V is spectrophotometer (UNICO S2100, Cole Parmer Instruments, Chicago, IL, USA). A standard curve was created using a standard glucose solution. Finally, total sugar content was expressed as mg glucose equivalents per g of fresh weight.

### Endogenous gibberellins

Leaf sample from treated trees were collected at flower bud induction period (OFF-Year). Samples were mixed with methanol and then vortexed in a falcon tube. The solution was then filtered using a 0.45 m syringe filter and put into a vial in a small volume. The quantification of GAs was performed by high performance liquid chromatography using Agilent 1260 infinity series system (Agilent, USA). The chromatographic conditions were column C18 (100 mm x 4.6 mm), detection wavelength 206 nm, injection volume of 20 µl and column temperature of 35 °C. Methanol-water (0.5% acetic acid) was used as the mobile phase, and the flow rate was 1 mL/min. Gibberellic acid (5 mg) was dissolved in 50 mL of methanol to have a stock solution (100 ppm). Stock solution is diluted to create a series of standard solutions of known concentrations (0.5, 1, 5, 10, 20, 40, and 80 ppm). A calibration curve is generated by plotting the peak area versus the standard concentrations. Concentration of GAs (µg/g FW) in leaf samples was determined from calibration curve using the corresponding peak area^5,29,30^.

### Cumulative impact of GA and CuSO_4_ in the following off year

In the following Off year; fruit set %, fruit drop %, number of fruits, fruit weight, and total yield were recorded to study the cumulative impact of the previous treatments on the following Off year on the same treated trees in the previous On year.

### Alternate bearing (AB) index

It was determined from the dividing difference between On year and the following Off year on the total yield of them^10^ as the following equation. Total yield includes all fruits which include regular and irregular fruit shape.

$$\text{Alternate bearing index}{\%}=\:\frac{\text{O}\text{n}\:\text{y}\text{e}\text{a}\text{r}\:\text{y}\text{i}\text{e}\text{l}\text{d}-\:\text{O}\text{f}\text{f}\:\text{y}\text{e}\text{a}\text{r}\:\text{y}\text{i}\text{e}\text{l}\text{d}}{\text{O}\text{n}\:\text{y}\text{e}\text{a}\text{r}\:\text{y}\text{i}\text{e}\text{l}\text{d}\:+\:\text{O}\text{f}\text{f}\:\text{y}\text{e}\text{a}\text{r}\:\text{y}\text{i}\text{e}\text{l}\text{d}}\times100$$

It was determined from the dividing difference between On year and the following Off year by the total yield of them^10^ as the following equation. Total yield includes all fruits which include regular and irregular fruit shape.

$$\text{Alternate bearing index}{\%}=\:\frac{\text{O}\text{n}\:\text{y}\text{e}\text{a}\text{r}\:\text{y}\text{i}\text{e}\text{l}\text{d}-\:\text{O}\text{f}\text{f}\:\text{y}\text{e}\text{a}\text{r}\:\text{y}\text{i}\text{e}\text{l}\text{d}}{\text{O}\text{n}\:\text{y}\text{e}\text{a}\text{r}\:\text{y}\text{i}\text{e}\text{l}\text{d}\:+\:\text{O}\text{f}\text{f}\:\text{y}\text{e}\text{a}\text{r}\:\text{y}\text{i}\text{e}\text{l}\text{d}}\times100$$

### Statistical analysis

The complete randomized design was used to experiment arrangement with three replicates; four trees were used per replicate. The collected data from the four trees was represented as individual experimental unit. The experiment layout involves randomly distributed treatments to experimental units. The selection of trees was presented in 12 rows. Each row includes a tree from each treatment. Each replicate consisted of four rows (a tree from each row). All treatments arranged in every position within each row (first, second, third and fourth order). Results were subjected to analysis of variance using the general linear model procedure—SAS software Version 9.0 (SAS Institute Inc., Cary, NC, USA). Duncan’s multiple range test^31^ at a significant level of *p* < 0.05 was used to compare the means between treatments.

## Results and discussion

### Fruit set % and fruit drop %

**Fig. 1:**
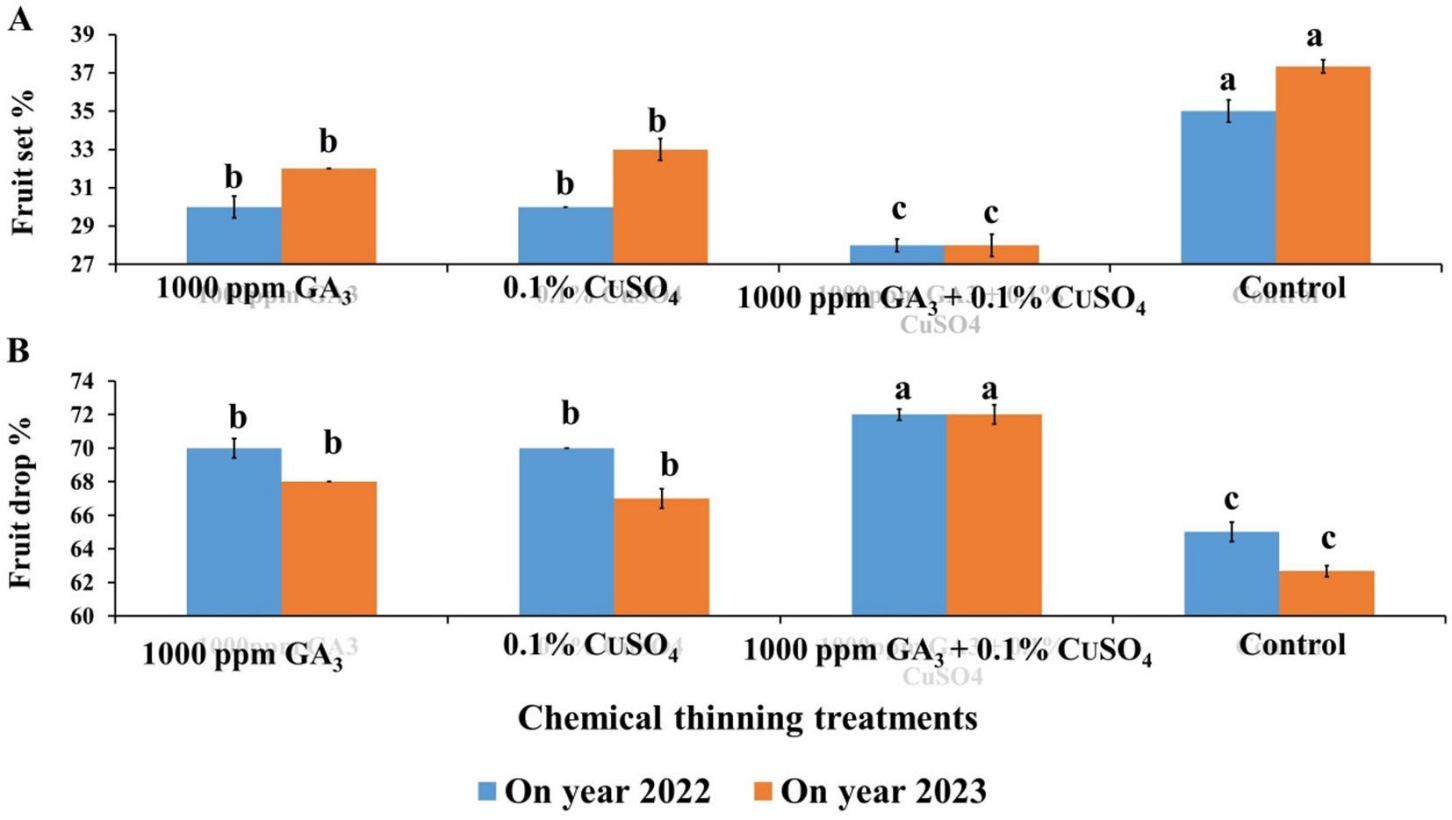
Effect of Chemical thinning treatments on fruit set % (A) and fruit drop % (B) of Balady mandarin trees during two On year seasons (2022&2023). Data are presented as means (*n* = 3 ± SE).

Individual application of GA or CuSO_4_ decreased fruit set % significantly compared to the control (Fig. 1A). Also, combined application of both GA and CuSO_4_ significantly decreased fruit set % and recorded the lowest percentage (25.33 & 28%) compared to all other treatments and control (35 & 37.3%). This reduction recorded about 27.6 and 25% compared to the control for both seasons. Individual application of GA or CuSO_4_ increased fruit drop % significantly compared to the control (Fig. 1B). Also, combined application of both GA and CuSO_4_ significantly increased fruit drop % and recorded the highest fruit drop % (74.67 & 72%) compared to all other treatments and control (65 & 62.67%). This increase in fruit drop % recorded about (14.88%) compared to the control for both seasons.

In this regard, exogenous GA (10 mg l^−1^) before bloom decreased fertilization and seed formation in self-incompatible Clemenules mandarin via reducing pollen growth and increased ovule abortion^22^. While, GA had no effect on seedless Satsuma mandarin^32,33^. In contrast, Mesejo et al.^22^ found that, GA application at 50 mg l^−1^ increased fruit set in seedless Clementine fruit. Also, GA treatments increased fruit set in some mandarin cultivars^33^. These results may be due to natural fruit drop happen for remove overlap fruit which trees cannot feeding it. So, chemical thinning particularly with high GA (1000 ppm) and CuSO_4_ (0.1%) concentrations resulted in rapid and early thinning which supplies photosynthetic resources and nutrients for the remaining fruitlets for proper time^34^.

Thinning treatment for flowers or fruits is necessary for producing high-quality fruits particularly for fruit trees species with extra fruit set such as citrus and peach which demand sufficient resources for fruit growth and development. In addition to decrease the alternate bearing phenomenon in these extra fruit set trees.

Spraying Balady mandarin trees by 200 ppm GA at anthesis stage (full bloom) or pre-anthesis stage (15 days before anthesis), or ovary stage at petal fall (15 days after anthesis) increased fruit thinning^11^.

### Number of fruits, fruit weight and fruit yield

**Fig. 2:**
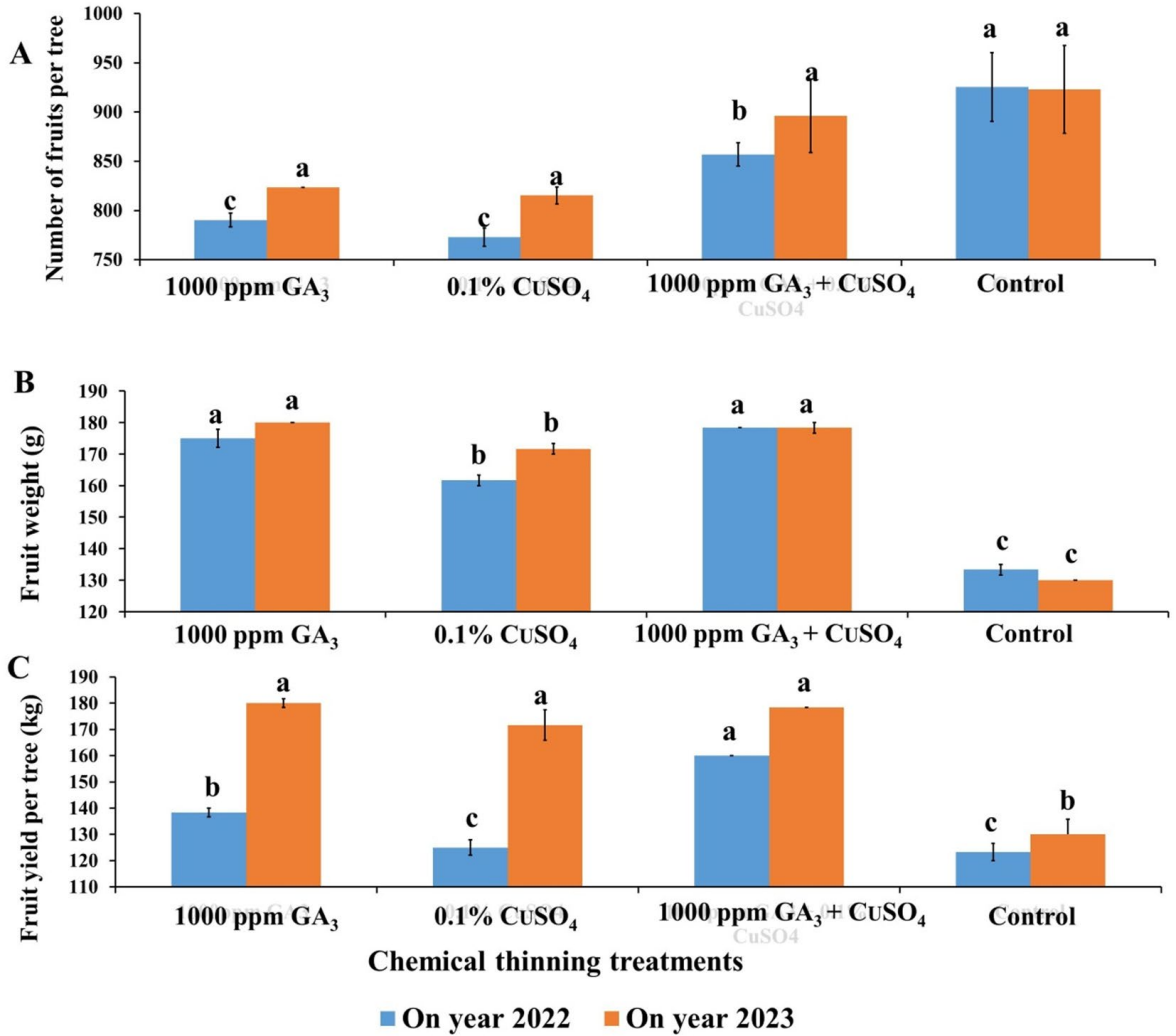
Effect of Chemical thinning treatments on number of fruits per tree (**A**), fruit weight (**B**) and fruit yield (**C**) of Balady mandarin trees during two On year seasons (2022 & 2023). Data were presented as means (*n* = 3 ± SE).

All chemical thinning treatments decreased fruits number per tree compared to the control (Fig. 2A). Also, CuSO_4_ recorded the lowest number of fruits per tree (773 & 815) with a reduction percentage of 16.46 and 11.66%, followed by GA (790 & 823) with about 14.59 and 10.76% reduction percentage in number of fruits compared to the control. The reduction in number of fruits due to GA application may be resulted from inhibiting fertilization^18–21^. While decreasing number of fruits due to CuSO_4_ application may be attributed to sulfur-based products which reduce pollen germination of the Fortune mandarin^24^. Also, pollen tube growth inhibition (94–100%) was recorded via sulfur application in Nadorcott mandarin flowers^25^.

All treatments significantly increased fruit weight compared to the control treatment (Fig. 2B). Also, GA alone (175 & 180 g) or GA + SO_4_ (175&178 g) recorded the highest fruit weights, which were higher than control (133 &130 g) by 31.25% & 38.46% and 31.25% & 37.17% for both treatments in both seasons, respectively.

The results of fruit yield per tree exhibited that, individual application of GA (138 & 148 kg per tree) or CuSO_4_ (125 & 150 kg per tree) significantly increased tree fruit yield per tree compared to the control (Fig. 2C). Moreover, these increases represent about 12% & 39% for GA and about 29% & 36% for GA + CuSO_4_ compared to the control (123 & 128 kg per tree) in both seasons, respectively.

Increasing fruit weight and yield due to GA and CuSO_4_ treatments were in agreement with El-Shereif et al.^20^ who found that, Balady mandarin sprayed with 25 ppm GA + Streptomycin at three times (beginning of flowering, 50% of full bloom and full bloom) recorded the highest fruit weight and yield. Also, Kheder et al.^11^ reported the treatments of 25 or 50 mg GA and 25 mg CuSO_4_ alone or in combination improved fruit weight and yield of treated Balady mandarin trees^12^. Moreover, spraying lime tree with 20 ppm GA or NAA (0, 10, 20, 30 ppm) improved fruit yield^35^. Furthermore, spraying Balady mandarin trees by 200 ppm GA_3_ at anthesis stage or pre-anthesis stage, or ovary stage at petal fall increased fruit thinning and the weight of remaining fruit and yield^11^. Combined application of 600 mg l^−1^ ZnSO_4_ + 400 mg l^−1^ CuSO_4_ five times, with the corresponding solution in one-month intervals beginning at full bloom (March to July), increased fruit yield up to 17% and improved fruit quality.

More recently, spraying Bac Son mandarin with GA (50, 75, 100, or 125 ppm) 7 days before flowering, full bloom, 7 days after full bloom or spraying CuSO_4_ (50, 75, 100, or 125 ppm) at 60% full bloom significantly increased fruit weight and yield^16^. Logically, early thinning during flowering provides more nutrients and carbohydrates for the remaining fruit which is more effective in increasing the weight of the remaining fruit than later fruit thinning as in hand thinning which consumes more nutrients and carbohydrates before removing it. So, it provides more nutrients and carbohydrates for more and bigger growth for the remaining fruit. On the other hand, application of 50 mg GA at full bloom reduced seedless fruit and yield of Afourer Mandarin^36^. While, GA application increased fruit set in seedless Clementine fruit, while it had no effect on Satsuma mandarins^31^.

### Normal and aborted seeds number

**Fig. 3:**
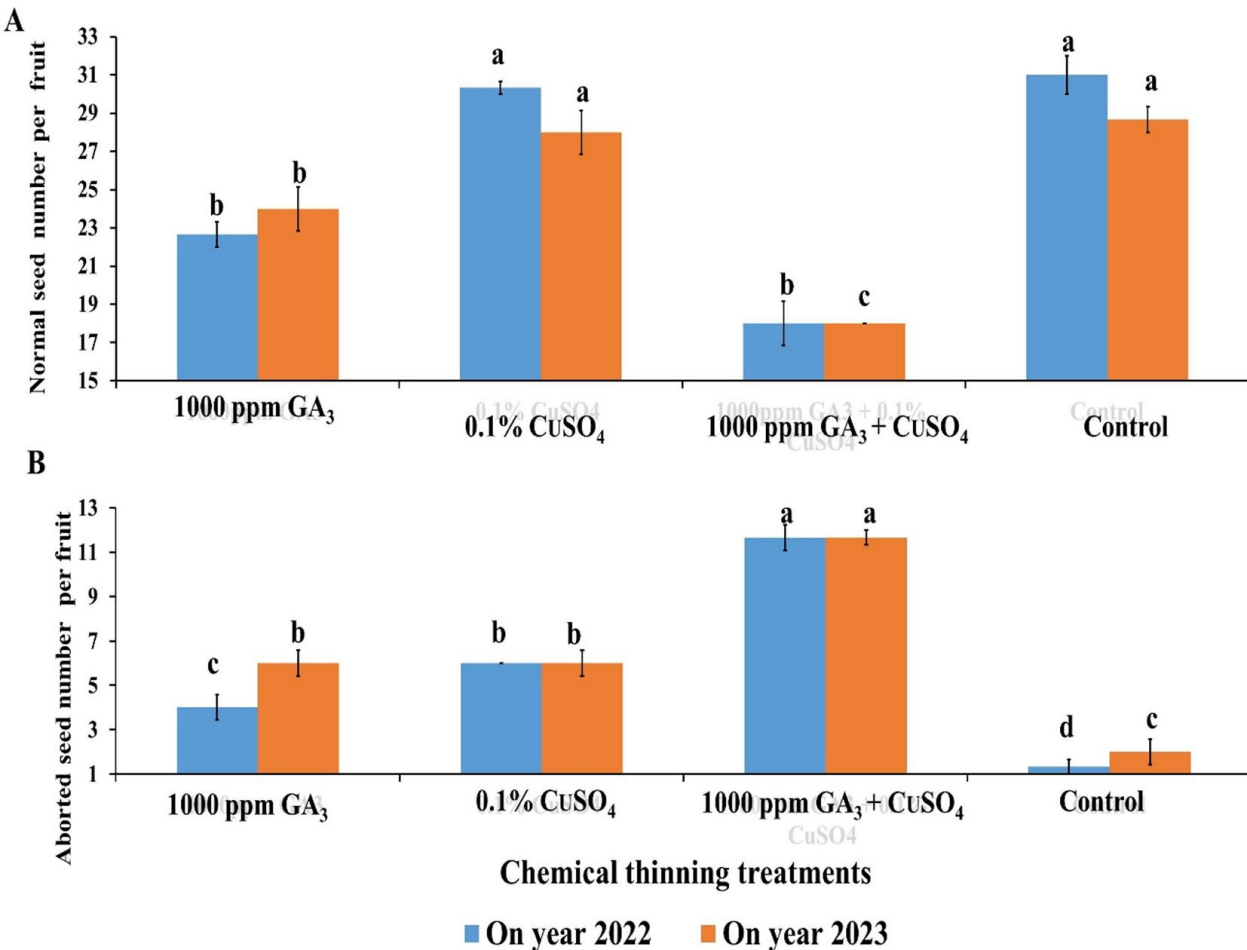
Effect of Chemical thinning treatments on normal number of seeds per fruit (**A**) and number of aborted seeds per fruit (**B**) of Balady mandarin fruit during two on year seasons (2022&2023). Data were presented as means (*n* = 3 ± SE).

**Fig. 4:**
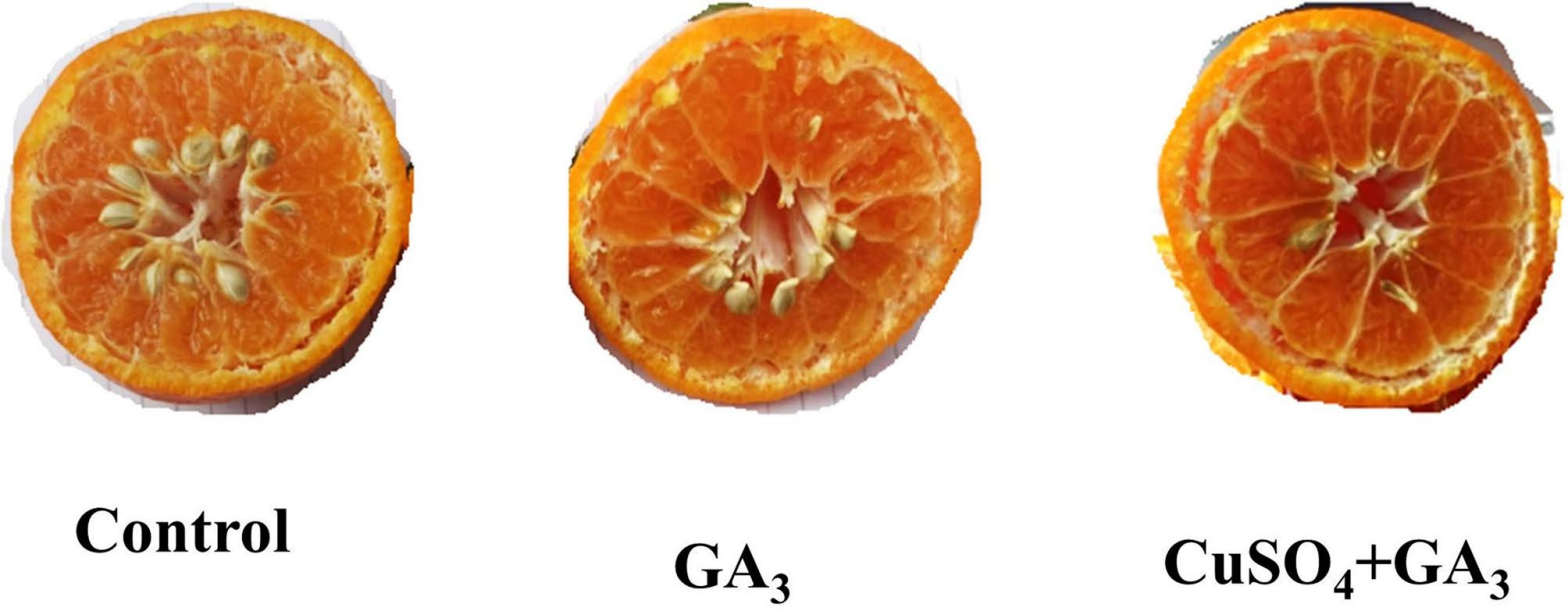
Effect of Chemical thinning (control, GA_3_, CuSO_4_) treatments on normal number of seeds per fruit.

The results of normal and aborted seeds number per fruit of treated mandarin trees by GA or CuSO_4_ and GA + CuSO_4_ were illustrated as shown in (Figs. 3A and 4). Application of GA significantly reduced the number of normal seeds per fruit (22.67 & 24) compared to the control (31 & 28.67) and this reduction percentage recorded 26.87% and 16.28%, respectively as shown in (Fig. 3A). Moreover, combined application of both GA and CuSO_4_ significantly recorded the lowest number of normal seeds per fruit (20 & 18) and this reduction percentage recorded 37.48% and 37.22% in the first and second seasons, respectively compared to the control.

On the other hand, the combined application of GA + CuSO_4_ significantly recorded the highest number of aborted seeds per fruit (10 & 11.67) and this increase recorded 651.8% & 483.5% in both seasons, respectively, compared to the control which had the lowest number of aborted seeds (1.33 & 2) per fruit (Fig. 3B). Meanwhile, the individual application of GA (4 & 6) or CuSO_4_ (6 & 6) significantly increased the number of aborted seeds per fruit by 200% & 200% and 351% & 200%, respectively, compared to the control.

GA has a vital role in fruit growth and seed formation^15^ and the quality of citrus fruit rely on physical (fruit weight) and chemical parameters (TSS/acid ratio) and low seed numbers per fruit^21,37^. Application of GA (10 mg l^−1^) before flowering decreased fertilization and seed formation in self-incompatible Clemenules mandarin via reducing pollen growth and increased ovule abortion^22^. In mandarin, spraying CuSO_4_ + GA increased floral style elongation which resulted in longer time for pollen tubes to reach the ovules and subsequently reduced ovules fertilization^21,22^. Balady mandarin sprayed with 25 ppm GA + Streptomycin at three times (beginning of flowering, 50% of full bloom and full bloom) reduced number of seeds per fruit by 76.6% and 77.4%^20^. Also, seeds inside the developing fruits exert strong inhibitory effect on flower bud production^10^. Spraying Balady mandarin trees by 200 ppm GA_3_ at full bloom gave the lowest number of seeds per fruit, which recorded 52.0% compared to the control treatment^11^.

Copper ions may may be having signalling of enzyme activity involved in seed formation^38,39^. More recently, application of 50 mg GA + 25 mg CuSO_4_ to Succarri orange trees increased the incomplete seed% and decreased the average number of seeds per fruit^8^. Foliar spray with 25 mg CuSO_4_ at 30% of full bloom of Yashar mandarin reduced number of seeds per fruit^40,41^. Sulphur succeeded in reducing number of seeds of Fortune mandarin via reducing pollen germination^24^. Also, 25 mg CuSO_4_ at full bloom increased seedless fruit of Clemenules mandarin and reduced number of seeds by 55–81%^24^. For effective practice to reduce number of seeds per fruit it must consider that, in citrus anthesis of single flower take 5–7 days and full bloom takes more than 20 days which show long overlapping stages among flower which make difficult to define specific time so spraying 3 times is more suitable^16^. CuSO_4_ (25 mg) at 60% of open flower decreased number of seeds in Afourer tangor fruits^24^. CuSO_4_ has a role in producing seedless fruit^23,39^.

The obtained results in the present study were in agreement with Hung et al.^16^ who found that, spraying Bac Son Mandarin with GA at 125 ppm (7 days before flowering, full bloom, 7 days after full bloom) or spraying CuSO_4_ (100, 125 ppm) at 60% of full bloom and at full bloom significantly reduced number of seeds per fruit. Both GA at 50 mg and CuSO_4_ at 25 mg reduced number of seeds of Afourer mandarin by 38%^18^. Number of seeds per Afourer mandarin reduced by 35% by GA or CuSO_4_^42^. Treatments of Balady mandarin by 25 and 50 mg GA and 25 mg CuSO_4_ alone or in combination reduced number of seeds per fruit with high efficiency for the combination treatment (4.6–7.6%)^12^.

### Marketable and misshapen fruit yield

**Fig. 5:**
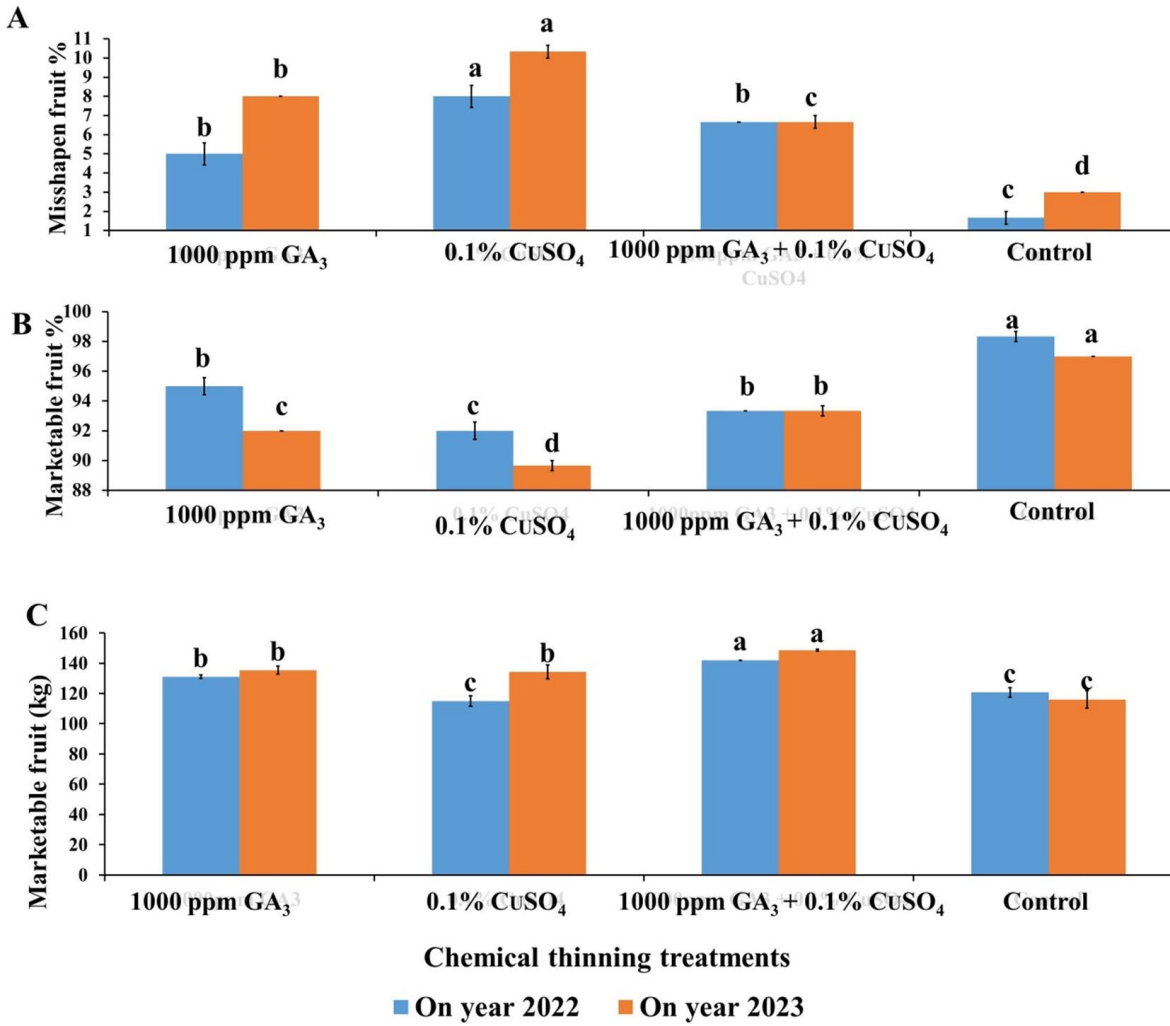
Effect of Chemical thinning treatments on misshapen fruit % (**A**), marketable fruit % (**B**) and marketable fruit (kg)/tree (**C**) of Balady mandarin fruits during two On year seasons (2022 &2023). Data were presented as means (*n* = 3 ± SE).

Individual application of CuSO_4_ significantly recorded the highest significant abnormal fruit shape (8% & 10%) followed by GA (5% & 8%) compared to the control in both seasons, respectively (Fig. 5A). Also, combined application of GA + CuSO_4_ significantly increased abnormal fruit shape by 5% & 6.7% in both seasons. Seeds which act as a source of GA, responsible for producing a normal fruit shape, while GA and CuSO_4_ treatments led to increasing aborted seeds percentage which led to increasing misshapen fruit percentage.

The data presented in Fig. 5B indicated that, control treatment recorded the highest marketable fruit percentage (98.3% & 97%) followed by GA (95% & 92%) and the combined application of GA + CuSO_4_ (93.41% and 93.45%) while CuSO_4_ recorded the lowest marketable fruit (92% & 89.3.

As shown in Fig. 5C, application of GA + CuSO_4_ recorded the highest total marketable fruits per tree (142–148.67 kg) followed by GA application (131–135.33 kg), while the control treatment recorded the lowest one (116–120 kg). The highest marketable fruit in GA + CuSO_4_ mainly resulted from increasing fruit yield.

### Fruit acidity, TSS and total sugars content

**Fig. 6:**
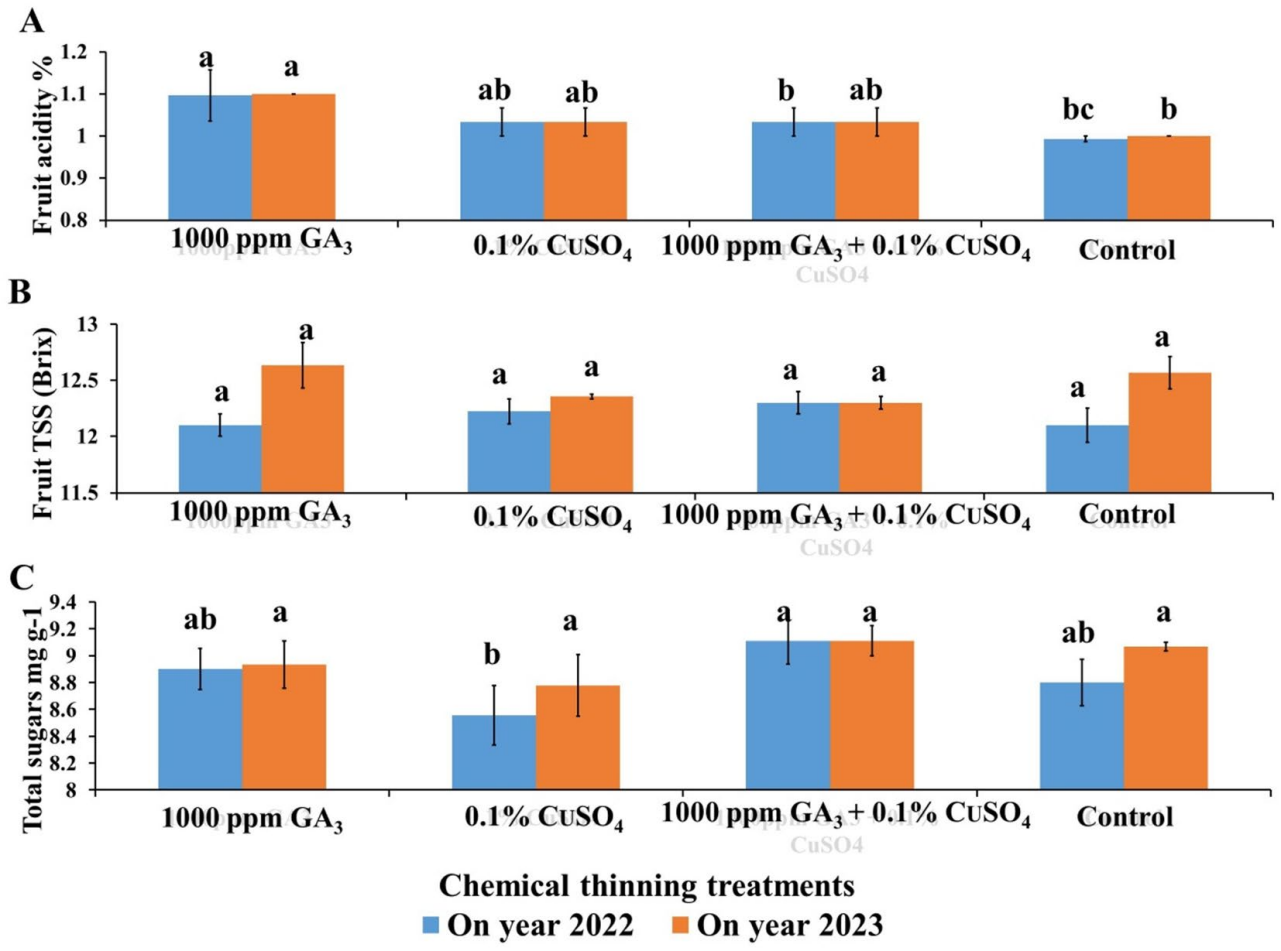
Effect of Chemical thinning treatments on fruit acidity % (**A**), fruit TSS (**B**) and fruit total sugars content (**C**) of Balady mandarin fruits during two On year seasons (2022 &2023). Data were presented as means (*n* = 3 ± SE).

GA treatment significantly recorded the highest fruit acidity % (1.10% & 1.10%) with an increase of 11.11% & 10% compared to the control which recorded 0.99% & 1% for both seasons (Fig. 6A). The difference between the other treatments were non-significant. Also, there were no significant differences between all treatments in fruit TSS (Fig. 6B). Combined application of GA + CuSO_4_ recorded the highest total sugars (9.31 mg g^−1^) with a significant value in the first season (Fig. 6C). Meanwhile, the differences between treatments were non-significant in general, this could be attributed to the reduction in fruits number due to chemical thinning treatments provide the nutrients and photosynthetic resources to increase fruit weight which resulted in higher yield compared to the untreated control.

In this regard, Balady mandarin sprayed with 25 ppm GA + Streptomycin at three times (beginning of flowering, 50% full bloom and full bloom) increased TSS/acid ratio^20^. Also, treatments of Balady mandarin by 50 mg GA + 25 mg CuSO_4_ improved fruit SSC/acid ratio^12^.

### Endogenous gibberellins

**Fig. 7:**
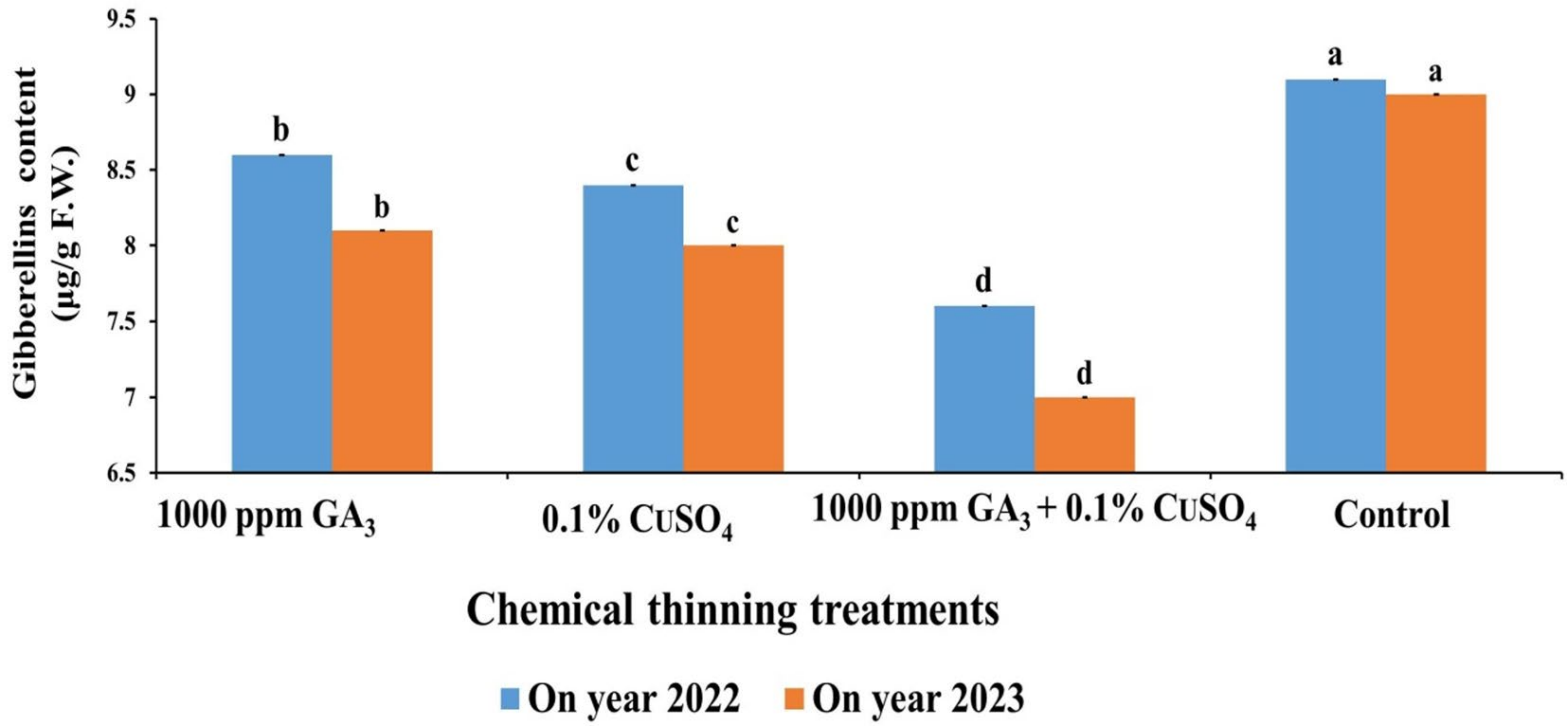
Effect of Chemical thinning treatments on gibberellins level during two On year seasons of Balady mandarin tree during two On year seasons. Data were presented as means (*n* = 3 ± SE).

The lowest endogenous GA level in leaves was recorded by GA + Cuso_4_ (7.6 &7U) by a reduction percent of 16.48 & 22.2% followed by CuSO_4_ (8.4 & 8 U) by a reduction percent of 7.69 & 11.1% then GA (8.6 & 8.1) by 5.49 & 10% compared to the control in both seasons, which recorded (9.1 & 9 U) the highest endogenous GA levels (Fig. 7).

In this regard, Shaban et al.^5^ observed a reduction in GA level in winter at flower bud induction time in Balady mandarin tree as indicators for more flowering and yield in the following Off year. Also, in Israel, application of 50 ppm GA during the flowering induction period (tree times during December) greatly reduces flower numbers^6^. Also, seeds inside the developing fruits exert strong inhibitory effect on flower bud production in apple^10^.

### Cumulative impact of chemical thinning in the following off year

**Fig. 8:**
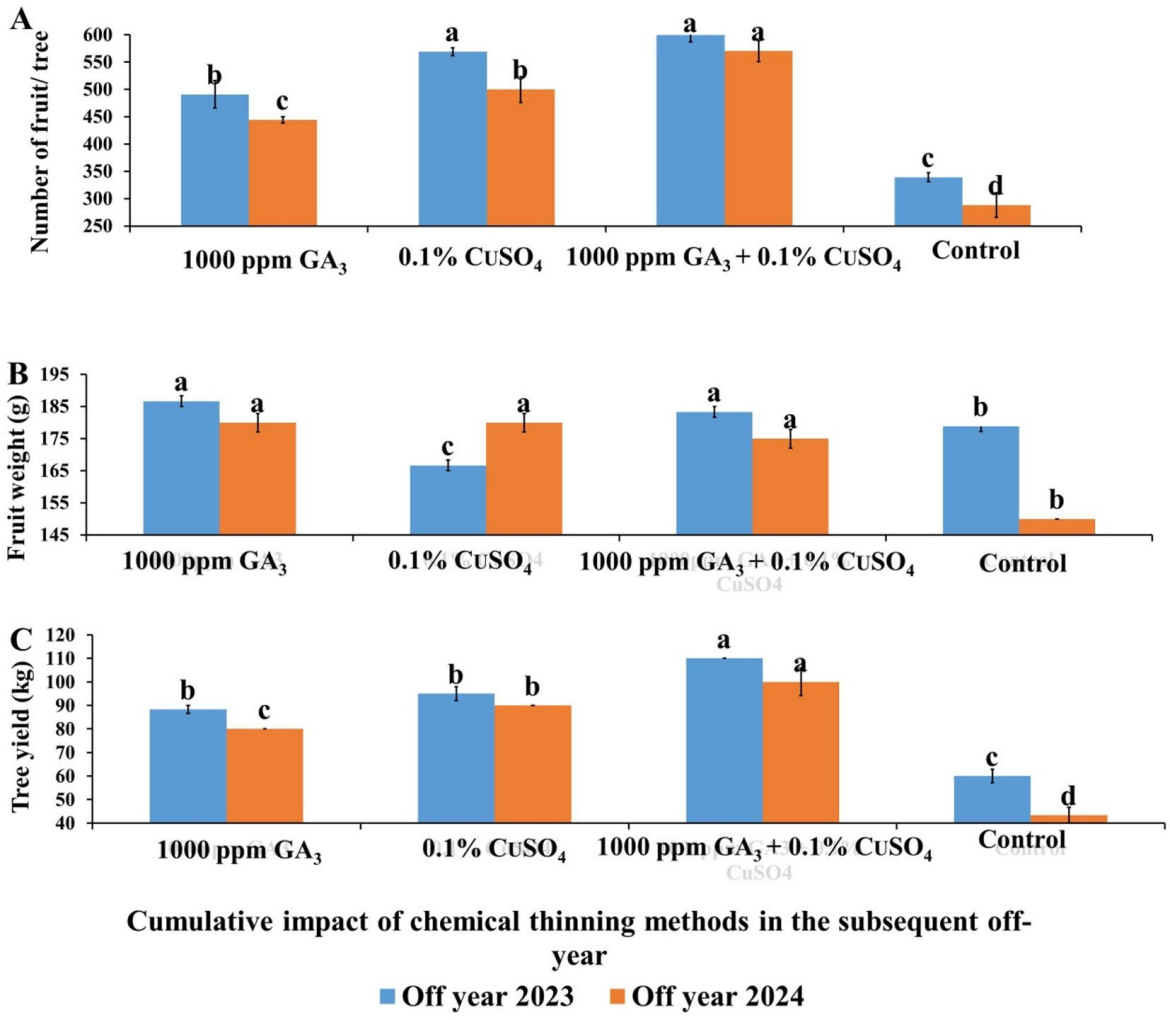
Effect of cumulative impact of chemical thinning treatments on number of fruits per tree (**A**), fruit weight (**B**) and fruit yield (**C**) of Balady mandarin trees during two Off year seasons (2023 &2024). Data were presented as means (*n* = 3 ± SE).

For the cumulative impact of chemical thinning treatments on the following Off year (Fig. 8), it could be concluded that, the cumulative impact of chemical thinning in the following Off year was observed on fruits number per tree (Fig. 8A). Thus, the treatment of GA + CuSO_4_ significantly recorded the highest fruits number per tree (599.67 & 570) followed by CuSO_4_ (569 & 500) and these results represent percentage of increases about (76.72% & 97.69%) and (67.78% & 73.41%), compared to the control (339.3 & 288.3) in both seasons, respectively.

The cumulative impact of chemical thinning in the following Off year on fruit weight was observed in Fig. 8B, it can be concluded that, GA alone (186.67 & 180 g) and GA + CuSO_4_ (183 & 175 g) gave the highest significant fruit weight with a percentage increase of 5.67%& 20% and 3.77%&16.67% for both treatments, compared to the control in both seasons respectively. Meanwhile, control gave the lowest values (176.67 & 150 g).

The cumulative impact of chemical thinning in the following Off year on fruit yield (Fig. 8C) indicated that, GA + CuSO_4_ gave the highest significant tree yield (110 & 100 kg per tree) with increases percent of 83% & 130% followed by CuSO_4_ (58.3% & 107.7%) for both treatments compared to the control (60 & 43.3 kg per tree) in both seasons, respectively. Increasing fruit yield in the following Off year resulted mainly from increasing number of fruits since the increase in number of fruits in GA + CuSO_4_ reached 83%& 130%, while the increase in fruit weight reached about 3.77% & 16.67% in both of the two Off years, respectively.

### Alternate bearing (AB) index

**Fig. 9:**
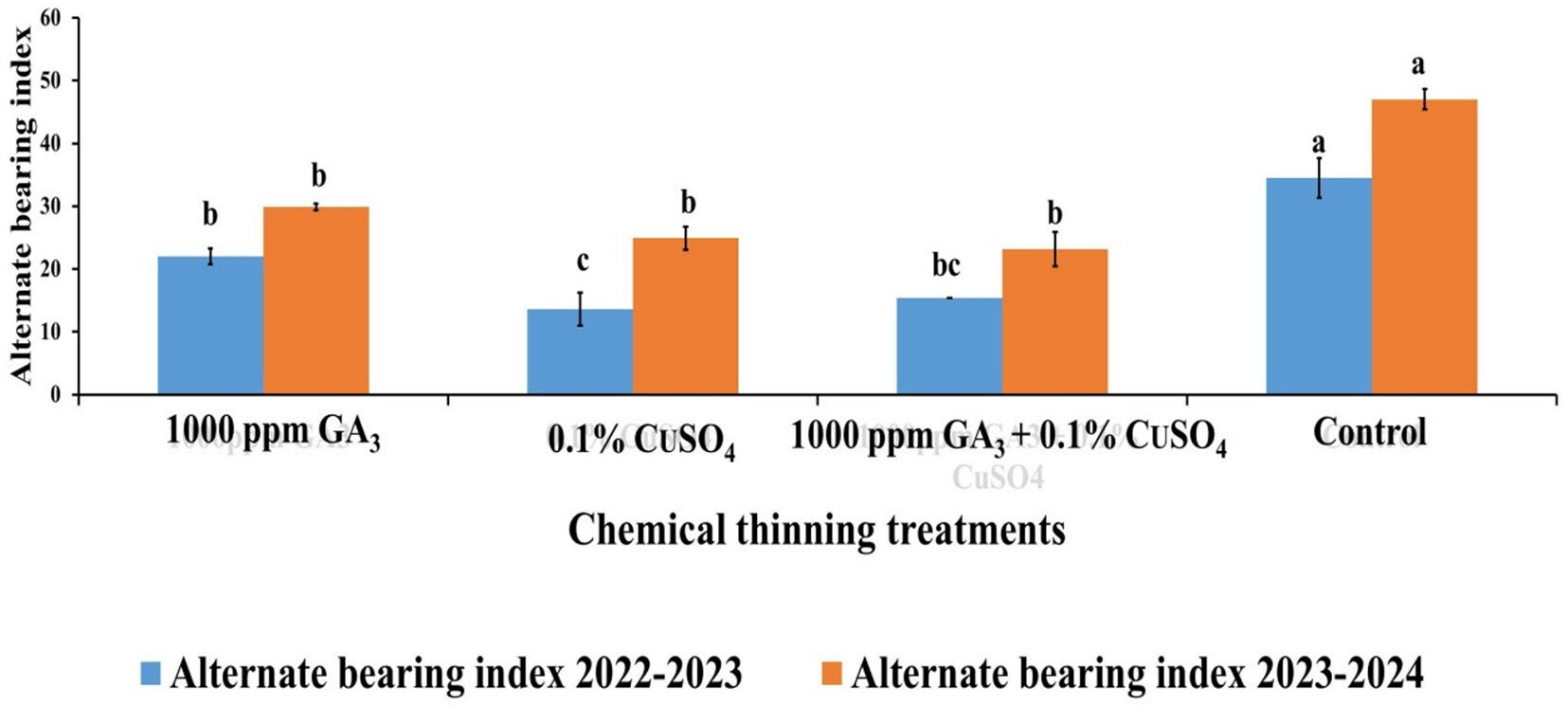
Effect of Chemical thinning treatments on alternate bearing index (B) between two On years and the following two Off years seasons of Balady mandarin tree during two On year seasons. Data were presented as means (*n* = 3 ± SE).

The recorded results of alternate bearing (AB) index % showed that, the control treatment recorded the highest AB % (34.51% & 47%) and both treatments CuSO_4_ (13.59% & 24.9%) and GA + CuSO_4_ (15.38% & 23.17%) recorded the lowest AB % with a high reduction percentage of 60.62% & 47% and 55.43% & 50.7%, while GA treatment recorded (22.04% & 29.87%) with a slight reduction percentage about 36% compared to the control in both seasons, respectively (Fig. 9).

Since Balady mandarin fruit contain more than 20 seeds per fruit^11,12^. Decreasing number of seeds which consider a contentious supplier with GA reduce endogenous GA level which led to improve flower bud induction in the winter prior to Off year and regulate bearing than control trees. Application of 50 ppm GA to Orri mandarin during the flowering induction period (tree times during December) greatly reduces flower number^6^. Since Balady mandarin more suspected to alternate bearing behaviour^5,7^. During On year, fruits produces different substances such as GA which is known as inhibitor for citrus flowering^13^. Seeds contain relatively large amounts of hormones (IAA & GA) that may act as an inhibitor to stimulate floral induction^14^. The treatment of GA + CuSO_4_ was more effective in reducing AB in Balady mandarin by decreasing number of seeds and number of fruits during the On season, which is considered one of the most GA supplier, and then it increased number of fruits in the following Off year. Also, this treatment increased yield during On season mainly by increasing fruit weight, while it increased fruit yield in the Off season by increasing number of fruits and fruit weight.

Finally, it could be concluded that the treatment of GA + CuSO_4_ is an effective chemical strategy for chemical fruit thinning in On year seasons, as a strategy for controlling AB in Balady mandarin. It is a low-cost effective method compared to the hand thinning method which requires more labor, costs and time. Also, this treatment is more efficient since it removes the flowers at an early stage, not removing small fruits that deplete more nutrients, water, carbohydrates and growth promotion substances. Thus, these substances sustain the growth of the retained flowers and fruits in GA + CuSO_4_ treated trees. Also, GA is commonly used in horticultural practices^6,12,15^ and Cu is an essential element as a nutrient with anti-fungal effects^23,39^. Moreover, regarding fruit quality in respect to the low number of seeds in the fruit, it is well known that hand thinning practices lead to produce more-seeded fruits which are less favorable (low quality fruits), on the other hand, the low-seeded fruits (high quality fruits) produced by the treatment of GA + CuSO_4_ are more favorable for customer demand.

## Conclusion

Individual application of 1000 ppm GA or 0.1% CuSO_4_ at 50 and 100% of flowering effectively decreased number of seeds per fruit, fruit set, and alternate bearing index in Balady mandarin. Moreover, the combined application of GA + CuSO_4_ recorded the lowest % of fruit set (27.6% & 25%), the lowest number of seeds/fruit (20 & 18) with the highest reduction percentage (35.48% & 37.22%) compared to the control (31 & 28.67), meanwhile, recorded the highest increase percentage of fruit weight (32.2% & 37.2%) and marketable yield (17.7% & 28.17%) compared to the control in the two On year seasons. Accordingly, GA + CuSO_4_ proved to be an efficient treatment which effectively reduced endogenous GA in leaves of mandarin trees, produced low-seeded fruits (high quality fruits), reduced AB index % and improved On year yield via increasing fruit weight and the following Off year yield via increasing fruits number per tree. Finally, it could be concluded that the treatment of GA + CuSO_4_ is an effective chemical strategy for chemical fruit thinning in On year seasons, as an efficient strategy for controlling AB in Balady mandarin.

## Data Availability

The data generated and/or analysed during the current study are available per request to the corresponding author.
